# Natural medicines of targeted rheumatoid arthritis and its action mechanism

**DOI:** 10.3389/fimmu.2022.945129

**Published:** 2022-08-01

**Authors:** Xueling Liu, Zhiguo Wang, Hua Qian, Wenhua Tao, Ying Zhang, Chunyan Hu, Weiwei Mao, Qi Guo

**Affiliations:** ^1^ School of Medicine, Jiangsu University, Zhenjiang, China; ^2^ Chinese Academy of Chinese Medical Sciences, Beijing, China; ^3^ Department of Traditional Chinese Medicine, Affiliated Hospital of Jiangsu University, Zhenjiang City, China

**Keywords:** rheumatoid arthritis, autoimmune disease, flavonoids, polyphenols, alkaloids, glycosides, terpenes, natural medicines

## Abstract

Rheumatoid arthritis (RA) is an autoimmune disease involving joints, with clinical manifestations of joint inflammation, bone damage and cartilage destruction, joint dysfunction and deformity, and extra-articular organ damage. As an important source of new drug molecules, natural medicines have many advantages, such as a wide range of biological effects and small toxic and side effects. They have become a hot spot for the vast number of researchers to study various diseases and develop therapeutic drugs. In recent years, the research of natural medicines in the treatment of RA has made remarkable achievements. These natural medicines mainly include flavonoids, polyphenols, alkaloids, glycosides and terpenes. Among them, resveratrol, icariin, epigallocatechin-3-gallate, ginsenoside, sinomenine, paeoniflorin, triptolide and paeoniflorin are star natural medicines for the treatment of RA. Its mechanism of treating RA mainly involves these aspects: anti-inflammation, anti-oxidation, immune regulation, pro-apoptosis, inhibition of angiogenesis, inhibition of osteoclastogenesis, inhibition of fibroblast-like synovial cell proliferation, migration and invasion. This review summarizes natural medicines with potential therapeutic effects on RA and briefly discusses their mechanisms of action against RA.

## Introduction

Rheumatoid arthritis (RA) is a common chronic inflammatory disease and systemic autoimmune disease, mainly involving the synovial joints, and is characterized by destroyed immune regulation of the joint synovial membrane, systemic inflammation and the existence of autoantibodies, which finally lead to severe damage and destruction of cartilage and bone (1, 2). At the inflammatory joint site, immune infiltration caused by cytokine and chemokine pathway disorders promotes the proliferation of joint fibroblast-like synoviocytes (FLSs) and leads to the development of chronic inflammation (2, 3). The disease will cause systemic symptoms such as fever, anemia, osteoporosis or muscle weakness. It will also affect other organs, such as the skin, blood vessels, kidneys, heart, lungs, nerves, liver and intestines and stomach (4, 5), and even increase the risk of patients suffering from cardiovascular diseases and certain cancers such as lung cancer and lymphoma (6, 7). Globally, the incidence of RA is approximately 1% (8). The pathogenesis of RA is mainly genetic factors, environmental factors and autoimmune (9). Smoking, hormones, infections, microbiota, etc. are the key factors leading to RA in genetically susceptible individuals (10, 11). At present, immunosuppressive drugs and non-steroidal anti-inflammatory drugs are commonly used in clinic to treat this disease, but these drugs have serious side effects. In order to alleviate the suffering of patients with RA and improve their quality of life, we need more kinds of clinical drugs that are more effective and have fewer side effects.

## Polyphenols

### Resveratrol

Resveratrol (RES) (3,5,4-trihydroxystilbene) is a natural antioxidant that exists in a variety of plants, such as Cassia obtusifolia, Veratrum nigrum, Polygonum cuspidatum, grapes, soybeans and nuts, and has a variety of pharmacological effects, as well as therapeutic effects on a variety of autoimmune diseases, including RA (12). A clinical study (13) has shown that RES can significantly improve the disease status of patients with RA and significantly reduce the serum levels of related biochemical indicators, namely, C-reactive protein, erythrocyte sedimentation rate, carboxylated osteocalcin, matrix metalloproteinase-3 (MMP-3) tumor necrosis factor α (TNF-α) and interleukin-6 (IL-6). The expression of silent information regulator 1 (SIRT1) in synovial tissue and FLSs of patients with RA is significantly lower than that in the healthy control group. SIRT1 is a key regulator of the pathogenesis of RA and plays an important role in the anti-inflammatory and anti-cytokine pathway of resveratrol in the inflammatory joint environment of RA (14). RES inhibits the NF-κB pathway by increasing the expression of SIRT1 and promotes FLSs apoptosis, reducing synovial hyperplasia and inflammatory response, thereby improving RA (15). This process may be mediated by RES by inhibiting the expression of MMP-1 and MMP-13 (16). Further research has shown that (17), NF-κB can directly bind to the promoter of SIRT1/nuclear factor erythroid 2-related factor 2 (Nrf2) signaling pathway and exert a negative regulatory effect. Lu et al. demonstrated that (18), resveratrol induced FLSs apoptosis through mitochondrial dysfunction and endoplasmic reticulum stress pathway. Fernández-Rodríguez et al. (14) showed that resveratrol could inhibit the proliferation of synovial tissue cells by inducing non-classical autophagy pathway and reducing the expression of p62.

The research by Buhrmann C et al. (19) showed that TNF-β induction could make chondrocytes produce a pro-inflammatory microenvironment similar to that of TNF-α and T lymphocytes, up-regulate the pro-inflammatory signaling pathway and inhibit the chondrogenic potential of chondrocytes, while resveratrol could inhibit the downstream signaling pathway of TNF-β, which might be achieved by inhibiting the NF-κB signaling pathway of chondrocytes and up-regulating the SIRT1 signal. Tian et al. (20) demonstrated that TNF-α induction could increase the production of IL-1β and MMP-3 inflammatory cytokines in RA-FLS, while resveratrol reduced the production of TNF-a-induced IL-1β and MMP-3 by inhibiting PI3K/Akt signaling pathway, thereby exerting the anti-inflammatory effect. The research by Tsai et al. (21) has revealed that particulate matter in air pollution can enhance the activity of nicotinamide adenine dinucleotide phosphate (NADPH) oxidase and reactive oxygen species (ROS) generation in human FLSs, enhance the activity of NF-κB pathway and activate Akt, ERK1/2 or p38 MAPK. However, resveratrol pretreatment can down-regulate the expression of cyclooxygenase (COX)-2 and its metabolite prostaglandin E2 (PGE2), inhibit oxidative stress, and reduce the activity of these inflammatory pathways. Studies have found that (22, 23), Src tyrosine kinase, Signal transducer and activator of transcription 3 (STAT3), mitogen-activated protein kinase (MAPK) and Wnt signaling pathways in collagen-induced arthritis (CIA) model are activated, and the level of hypoxia-inducible factor-1α (HIF-1α) in RSC-364 cells stimulated by IL-1β is increased. Resveratrol can mediate angiogenesis in synovial tissue by inhibiting these signaling pathways and HIF-1α, with a preventive role in the progression of RA. Theoretically, resveratrol can play a role in improving RA by resisting inflammation, oxidation, inhibiting cell proliferation, promoting apoptosis in synovial tissues and inhibiting angiogenesis. Resveratrol can be considered as a new therapeutic drug for RA, with great potential.

### Epigallocatechin-3-gallate

Epigallocatechin-3-gallate (EGCG), a catechin monomer extracted from tea leaves, is the main active component of tea polyphenols and has antioxidant and anti-inflammatory effects (24). Studies (25, 26) have shown that EGCG has an anti-inflammatory effect in human RASFs, and is the best anti-inflammatory catechin in green tea extract, which can inhibit the expression of IL-1β-induced chemokines (ENA-78, growth related gene α and reduced upon activation, nornal T cell expressed and secreted), cytokines (TNF-α, IL-6 and IL-8), COX-1, COX-2, MMP-1 and MMP-2. According to the research by Karatas et al. (27), EGCG can improve the symptoms of arthritis in rat CIA model, which may be achieved by reducing the level of pro-inflammatory cytokines (IL-17 and TNF-α), regulating the transcription factor Nrf2 and the balance between the induced inflammation and oxidation-oxidation resistance. Studies (28, 29) have shown that EGCG can reduce the symptoms of autoimmune arthritis, inhibit osteoclastogenesis and Th17 cell activation, and increase the number of Foxp3^+^ Treg. Its anti-arthritis effect may be due to the induction of pERK, Nrf2 and heme oxygenase-1 (HO-1) expression, and inhibition of STAT3 activation. Further studies have shown that EGCG inhibits Th17 differentiation by inhibiting STAT3 activation, and also inhibits mTOR and subsequent activation of HIF-1α, which may lead to the reduction of Th17 and enhancement of Treg. These studies suggest the potential value of EGCG in the treatment of RA.

### Curculigo glycoside

Curculigo glycoside is the main saponin active substance in Curculigo orchioides and has significant antioxidant and anti-osteoporosis effects (30). A study (31) has shown that Curculigoside A (CA) can improve the symptoms of arthritis in adjuvant-induced arthritis (AIA) rats, which may be mediated by reducing the expressions of pro-inflammatory factors (IL-6, IL-1β, TNF-α) and PGE2, regulating the oxidation-oxidation balance, and down-regulating the NF-κB/NLRP3 pathway. Tan’s research shows that (32), curculigo glucoside has a significant anti-arthritis effect, which can significantly inhibit the proliferation of MH7A cells, improve the arthritis symptoms of type II collagen-induced arthritis (CIA) rats and reduce the levels of inflammatory factors (TNF-α, IL-1β, IL-6, IL-10, IL-12 and IL-17A). Its anti-arthritis molecular mechanism may be related to the JAK/STAT/NF-κB signaling pathway. According to network pharmacology research (33), *EGFR*, *MAP2K1*, *MMP2*, *FGFR1* and *MCL1* are potential target genes for CA to treat RA. CA may inhibit the expression of these genes and exert anti-RA effects by acting on nitrogen metabolism, estrogen signaling pathway, RAS-associated protein 1 (Rap1) signaling pathway, PI3K/Akt signaling pathway, etc.

## Flavonoids

### Icariin

Icariin (ICA), the active monomer of flavonoid glycosides extracted from Epimedium grandiflorum, has the pharmacological activities of anti-oxidation, anti-inflammation, anti-tumor, regulating sex hormones, alleviating atherosclerosis, etc. It also has a certain effect on autoimmune diseases such as RA, bronchial asthma, multiple sclerosis and systemic lupus erythematosus (34). A study (35) showed that icariin could inhibit the cartilage and bone degradation of CIA mouse model, and had cathepsin K activity. Chi et al. (36) suggested that icariin could alleviate RA by inhibiting the expression of osteoclast markers (β3 integrin, cathepsin K and MMP-9), reducing the number of Th17 cells, and inhibiting STAT3 activation-mediated IL-17 production. Further research (37) has shown that the inhibition of joint bone loss and the reduction of joint damage by ICA are mediated by lower nuclear factor ξ β ligand receptor activator (RANKL) and higher OPG expression. Wu et al. (38) demonstrated that ICA could inhibit the proliferation of RA-FLS and the secretion of inflammatory cytokines (TNF-α, IL-1β and IL-6), and promote the apoptosis of RA-FLS by up-regulating the miR-223-3p/NLRP3 signaling pathway. miR-223-3p may be a potential therapeutic target for ICA to alleviate RA. Synovitis is an important pathological process of RA. Luo et al. (39) studied the effect of ICA on synovitis using a lipopolysaccharide (LPS)-induced synovitis cell model. The results showed that ICA could inhibit iron ptosis by activating the Xc-/glutathione peroxidase 4-axis, thereby reducing the mortality of LPS-induced synovial cells and protecting synovial cells. These studies indicate that ICA is a promising agent for the treatment of RA and synovitis.

### Apigenin

Apigenin is a dietary flavonoid compound widely distributed in fruits and vegetables, and has multiple biological activities such as anti-inflammation, anti-oxidation, and pro-apoptosis (40, 41). Studies (42, 43) have shown that apigenin has a pro-apoptotic effect in human RA-FLS, and it can induce RA-FLS apoptosis by mediating ROS and oxidative stress activator ERK1/2, and mediate apoptotic cell death by activating the apoptotic effector caspase-3/7. Tumor necrosis factor-related apoptosis-inducing ligand (TRAIL) induces apoptosis in human RAFLS and subsequently induces the proliferation of viable cells. Apigenin, in turn, promotes TRAIL-induced apoptosis in human RA-FLS and inhibits TRAIL-dependent proliferation of RA-FLS by increasing the expression and activity of caspase-3, decreasing the ratio of Bcl-2/Bax, restoring the expression of cell cycle inhibitors p21 and p27, and activating the PI3K/AKT signaling pathway. The research by Chang et al. (44) has shown that apigenin can improve the symptoms of arthritis in CFA rats and its anti-inflammatory mechanism may be mediated by reducing the expression of cytokines (IL-1β, IL-6 and TNF-α) and inhibiting the P2X7/NF-κB signaling pathway. Li et al. (45) showed that apigenin could prevent arthritis in CIA mice and might be a potential therapeutic drug for arthritis. *In vitro*, apigenin inhibits LPS-stimulated maturation and chemotaxis of bone marrow-derived dendritic cells (BMDC) by inhibiting the expression of costimulatory molecules (CXCR4 and CCR7) and MHCII and reducing the secretion of cytokines (TNF-a, IL-12p70 and IL-10). *In vivo*, apigenin reduces the secretion of pro-inflammatory factors (IL-1β, IL-6, and TNF-α), reduces the expression of the DCs costimulatory molecules CXCR4 and MHCII, and reduces the number of Langerhans cells. The above studies have shown that apigenin can play an anti-RA role by resisting inflammation and promoting apoptosis, and inhibiting the maturation and migration of DCs.

### Quercetin

Quercetin, a natural flavonoid compound, is widely distributed in herbs, vegetables and fruits and has a variety of pharmacological effects. In addition, it plays a role in improving the clinical symptoms of RA, alleviating inflammation and preventing the formation of pannus, so that quercetin can be used as a natural drug to assist in the treatment of RA (46, 47). A study (48) showed that quercetin could inhibit RANKL production and monocyte-to-osteoclast formation in IL-17-stimulated RA-FLS, inhibit Th17 differentiation and IL-17 secretion, and exert immunomodulatory effects in IL-17-stimulated RA-FLS through mTOR, ERK and NF-κB pathways. Yang et al. (49) showed that quercetin could reduce the symptoms of arthritis in CIA rats, and its potential mechanism might be mediated by regulating Th17/Treg balance, inhibiting the activation of NLRP3 inflammasomes and activating HO-1-mediated anti-inflammatory response. Endale et al. (50) further studied the molecular mechanism of quercetin against RA. The results showed that the anti-inflammatory effect of quercetin on LPS-induced RAW264.7 cells was achieved by inhibiting the production of MAPK/activating protein-1 (AP-1) and IKK/NF-κB-mediated inflammatory mediators (TNF-α, IL-1β, IL-6, and macrophage colony stimulating factor) and the formation of TLR4/myeloid differentiation factor-88 (MyD88)/PI3K complex. These studies have shown that quercetin exerts anti-RA effects mainly through anti-inflammation, immune regulation and inhibition of osteoclasts formation.

### Baicalin

Baicalin is a flavonoid active compound extracted from the root of Scutellaria radix. Previous study (51) has shown that baicalin has an anti-inflammatory effect in RA-FLS and inhibits IL-1β-induced proliferation of RA-FLS, which is related to its inhibition of NF-κB transcriptional activity and macrophage migration inhibitory factor (MIF)-mediated MAPK/ERK/p38 signaling pathway. A study (52) has shown that baicalin can improve joint inflammation in CIA mice, inhibit the expansion of Th17 cells in the body, and down-regulate the expression of adhesion molecules (ICAM-1 and VCAM-1) and inflammatory factors (IL-6 and TNF-α) in synovial cells stimulated by IL-17. The research by Wang et al. (53) showed that baicalin treatment could reduce joint inflammation in CIA rats, which was related to that baicalin could inhibit the expression of NF-κB p65 protein in synovial tissue and FLSs, and down-regulate NF-κB p65 acetylation by increasing Sirt1. Studies (54, 55) have shown that the anti-RA effect of baicalin is related to the down-regulation of pro-inflammatory factors (TNF-α, IL-1β, and IL-6) and inflammatory markers (MMP-2, MMP-9, iNOS, and COX-2), the induction of monocyte apoptosis in synovial fluid of CIA mice, and the inhibition of JAK1/STAT3 and TLR2/MYD88/NF-κB p65 signal transduction. The above studies have shown that Baicalin has anti-RA activity.

## Alkaloid

### Sinomenine

Sinomenine is the main active ingredient isolated from the Chinese medicinal Caulis Sinomenii for the treatment of rheumatic diseases, and has the biological effects of anti-inflammation, anti-oxidation, inhibition of apoptosis, immunosuppression, etc (56–58). Clinical studies have shown that (59, 60) sinomenine has a significant effect on the treatment of RA. The study of sinomenine in RA-FLS by Liao et al. has shown that it has an antioxidant effect in anti-RA (61). Sinomenine can phosphorylate p62 ^Ser351^ to degrade Keap1 and increase Nrf2 expression, and play a role in protecting bone destruction by increasing p62 expression and activating the p62-Keap1-Nrf2 axis through p62 ^Thr269/Ser272^ phosphorylation. Studies (62, 63) have shown that sinomenine can inhibit the levels of inflammatory factors (TNF-α, IL-6, NO, PGE2, iNOS and COX-2) in IL-1β-induced RA-FLS, and inhibit the expression of TLR4, MyD88, p-NF-κB p65 and TRAF-6 in RA-FLS, suggesting that sinomenine prevents IL-1β-induced inflammation in human RA-FLS by inhibiting the TLR4/MyD88/NF-κB signaling pathway. The research by Zeng et al. (64) showed that sinomenine could inhibit the LPS-induced immune response of macrophages by down-regulating the levels of inflammatory cytokines (TNF-α, IL-1β and IL-6) and blocking the activated TLR4/NF-κB signaling pathway, indicating that sinomenine had an immune regulation effect in RA, which was consistent with the former research.

Zhou et al. demonstrated that (65), the mechanism of sinomenine improving inflammation and arthritis is partly related to the inhibition of microsomal prostaglandin E synthase 1 expression by reducing the DNA binding capacity of NF-κB. According to the research by Tong et al. (66), the mechanism of sinomenine inhibiting arthritis in CIA rats may be related to regulating the frequency of Treg and Th17 cells in intestinal lymph nodes and transporting lymphocytes (especially Treg cells) from the intestine to the joints. Feng et al. (67) showed that sinomenine could reduce the arthritis of CIA mice by inhibiting angiogenesis, and the mechanism might be related to the HIF-1α/vascular endothelial growth factor (VEGF)/angiopoietin 1(ANG-1) axis. α7 nicotinic acetylcholine receptors (α7nAChR) are key receptors for inhibiting inflammation in the cholinergic anti-inflammatory pathway, and there is a correlation between them and RA, while sinomenine can inhibit the expression of α7nAChR and exert anti-inflammatory and anti-arthritis effects through ERK/Egr-1 signaling pathway transduction (68, 69). These studies have shown that sinomenine can exert anti-RA effects through a variety of pathways, including anti-oxidation, anti-inflammation, immune regulation, and inhibition of angiogenesis. Therefore, sinomenine can be selected as one of the options for medical treatment of patients with RA.

### Norisoboldine

Norisoboldine (NOR), the main isoquinoline alkaloid present in the dried roots of Radix Linderae, may have certain anti-RA activity (70, 71), which can improve synovitis and abnormal immune status of CIA mice, and inhibit the activation of RAW264.7 macrophages by down-regulating the MAPKs signaling pathway. The research by Lu et al. (72) showed that NOR could inhibit the angiogenesis of synovial membrane in AIA rats, not only reducing the number of blood vessels and the expression of growth factors in the synovial membrane, but also inhibiting the migration and production of endothelial cells *in vitro*. The molecular mechanism of anti-angiogenesis was related to the activation of Notch1 signaling pathway by binding to Notch1 transcription complex. Wei et al. (73) believed that the important mechanism for NOR to exert anti-RA characteristics might be to prevent the release of IL-6 from FLSs, and its mechanism might be related to the inhibition of PKC/MAPKs/p65/cAMP response element-binding protein (CREB) pathway. Luo et al. (74) found that NOR had a pro-apoptotic effect on FLSs of AIA rats, and its molecular mechanism was achieved by promoting the release of cytochrome C, regulating the Bax/Bcl-2 expression-mediated mitochondrial dependent pathway, and up-regulating the p53 pathway. Studies (75, 76) have shown that NOR can inhibit the destruction of bone and cartilage in AIA rats by reducing the expression of RANKL, IL-6, PGE2 and MMP-13 through the p38/ERK/AKT/AP-1 pathway. The molecular mechanism may be achieved by inhibiting the ubiquitination of TNF receptor associated factor 6 (TRAF6), the aggregation of TRAF6-TAK1 complex and the activation of MAPKs/NF-κB/c-Fos/nuclear factor of activated T cell cytoplasmic 1 (NFATc1) pathway to prevent the differentiation and function of osteoclasts. In order to further study the anti-arthritis mechanism of NOR, Fang et al. (77) used non-targeted metabolomics to analyze the endogenous metabolites in urine of CIA rats and finally screened out 22 differential metabolites, most of which were related to lipid metabolism. Besides, it was also found that NOR could up-regulate the expression of carnitine acyltransferase 1 and down-regulates the expression of fatty acid synthase, suggesting that they may be new targets of NOR for the treatment of RA. These studies have shown that NOR is an effective monomer drug for the treatment of RA, which can play a role by anti-inflammation, immune regulation, promoting synovial cell apoptosis, inhibiting osteoclast differentiation and synovial angiogenesis, and regulating lipid metabolism.

Aryl hydrocarbon receptor (AhR) is a transcription factor that regulates ligand activation of foreign body metabolic disease (78). Prior to ligand binding, AhR was present as a complex in the cytoplasm. When AhR was bound to its agonist, AHR was dissociated from the protein complex and translocated to the nucleus, where it interacted with aryl hydrocarbon receptor nuclear transporter (ARNT) to form a dimer, and activated transcription of target genes by binding to specific enhancer sequences in the regulatory regions of target genes, such as the aryl hydrocarbon receptor response element on the cytochrome P450 Family 1 subfamily B member 1 (CYP1B1) promoter (78). A study (79) has found that AhR is highly expressed in the early stage of osteoclast formation, but its expression is reduced in mature osteoclasts, participating in human osteoclast differentiation. AhR may be a target molecule for preventing bone destruction in chronic inflammatory diseases such as RA. It is currently believed that AhR signaling pathway plays a key role in inflammatory diseases and autoimmune diseases (80). Studies (81, 82) have revealed that NOR can stably bind to AhR, promote AhR/Hsp90 dissociation and AhR nuclear translocation, increase the accumulation of AhR-ARNT complex, activate AhR-mediated reporter gene and up-regulate the expression of CYP1A1. In addition, NOR also inhibited the reduction of AhR/NF-κB/p65 complex caused by nuclear translocation of NF-κB p65 in osteoclasts, and reduced the expression of VEGF, resulting in the reduction of accumulation of ARNT/HIF-1α complex, suggesting that NOR reduced osteoclast differentiation and bone erosion by activating AhR and inhibiting the subsequent NF-κB and HIF pathways (81). NOR promoted the differentiation and function of Treg cells in intestinal tissue of CIA mice (in an AhR-dependent manner) by activating AhR, and thus played an anti-arthritis role (82). AhR antagonists such as resveratrol could largely reverse the effect of NOR, which was the agonist of AhR. The role of AhR in the immune system and potential clinical value of AhR antagonists in the treatment of RA (83).

### Tetrandrine

Tetrandrine, a dibenzyl isoquinoline alkaloid isolated from the root of the anti-rheumatic Chinese medicinal Stephania tetrandraS. Moore, has been proved to have medicinal potential for the treatment of RA (84). Studies (85, 86) have shown that the anti-inflammatory mechanism of tetrandrine in the treatment of RA may be related to its efficacy in inhibiting the LPS-induced phosphorylation of IκBα and NF-κB p65 in RAW 264.7 macrophages and ATDC5 chondrocytes and reducing the expression of COX in peripheral blood mononuclear cell of CFA rats. A recent study (87) has also shown that tetrandrine can improve RA by regulating neutrophil activity in AIA mice and reducing the formation of neutrophil extracellular traps induced by phorbol ester. AhR is essential for the differentiation and activation of Th17 cells (88). Tetrandrine, as a potential ligand of AhR, can activate AHR and regulate the Th17/Treg balance to inhibit osteoclastogenesis and improve arthritis in CIA rats (89). Tetrandrine can enhance the ubiquitination and degradation of spleen tyrosine kinase (Syk) through AhR/non-receptor tyrosine kinase/c-Cbl signaling pathway and regulate the expression of Syk and phospholipase-Cγ, thereby inhibiting osteoclastogenesis and bone destruction in arthritis (90, 91). C-src, as a binding protein of AhR, can bind to AhR in the cytoplasm. Once AhR is activated by ligands, c-src will be released and activated (92), followed by c-Cbl, which may also be activated as a substrate of c-src. Lv et al. showed that (93), tetrandrine can down-regulate the expression or activation of Akt/JNK, MMP-2, MMP-9, and fibrous F-actin and focal adhesion kinase FAK, reduce the expression of migration-related proteins Rac1, Cdc42 and RhoA in MH7A cells, and block the migration and invasion of RA-FLS. These studies have shown that tetrandrine can be used as a potential drug for the treatment of RA.

## Terpenoids

### Triptolide

Triptolide, a diterpenoid epoxide separated from Tripterygium wilfordii Hook. F., has strong anti-inflammatory effect and anti-RA activity (94), and can significantly inhibit TNF-α-induced gene expressions of IL-1β, IL-6, and IL-8 in MH7A cells, and induce apoptosis in MH7A cells. In the research by Wen et al. (95), the overexpression of ENST 000060619282 increases the levels of pro-apoptotic and pro-inflammatory factors, and reduces the levels of anti-apoptotic proteins and anti-inflammatory factors at the same time. However, the mechanism of triptolide in promoting the apoptosis of RA-FLS cells and reducing the inflammatory response may be achieved by down-regulating ENST 00000619282 (lnc RNA). Studies (96–98) have shown that triptolide can inhibit the proliferation, migration and invasion of FLSs. Triptolide can inhibit LPS-induced FLSs migration and invasion by inhibiting TLR4/NF-kB pathway-mediated MMP-9 expression (96), and the molecular mechanism for inhibition of MMP-9 is to inhibit MMP-9 transcriptional activity by inhibiting NF-kB binding activity in the MMP-9 promoter. Triptolide also reduced F- actin polymerization, which was related to the inhibition of MAPK signaling pathway activation (reducing TNF-α-induced phosphorylation of JNK) (97). Another study (98) has shown that triptolide inhibits the proliferation of FLSs and the expression of inflammatory cytokines (IL-1β, IL-6, and VEGF) induced by IL-6/sIL-6R complex by inhibiting the JAK2/STAT3 signaling pathway. Huang et al. (99) showed that triptolide could improve RA by down-regulating the inflammatory function of neutrophils (inhibiting the expression of pro-inflammatory cytokines, inhibiting migration, NETosis and autophagy, and promoting apoptosis). Kong et al. (100) found that Triptolide could inhibit angiogenesis of RA, down-regulate the expression of angiogenic activation factors (TNF-α, IL-17, VEGF, VEGFR, ANG-1, ANG-2 and Tie2), and inhibit the activation of MAPK signaling pathway (phosphorylation of ERK, p38 and JNK). These studies have shown that triptolide can exert anti-RA activity through a variety of pathways and can be used as a potential therapeutic agent for RA.

### Geniposide

Geniposide (GE) is a iridoid glycoside compound isolated from Gardeniae Fructus, the dried ripe fruit of Gardenia jasminoides Ellis. Studies (101, 102) have shown that GE has a certain anti-arthritis effect, probably by inducing Th17 cell immune tolerance and down-regulating p-JNK expression in mesenteric lymph node lymphocytes (MLNL) and peripheral blood lymphocytes (PBL) of AIA rats to enhance Treg cell-mediated activity, to exert anti-inflammation and immune regulation. Further study (103) has revealed that its anti-inflammatory and immunomodulatory effects may be related to the inhibition of the activation of MAPK signaling pathways (JNK, ERK1/2 and p38). The conversion of activated Gα protein subunits (Gαs)/inhibited Gα protein subunits (Gαi) can be coupled to sphingosine-1-phosphate receptor (S1PRs), which induces the activation of pro-inflammatory signals in RASFs by binding to S1PRs, thus playing a key role in RA. GE could inhibit the process (104), thereby inhibiting the abnormal proliferation, migration and invasion of MH7A cells, as well as the release of inflammatory factors, the expression of cAMP and the activation of ERK protein. Studies (105, 106) have found that GE also has a role in preventing angiogenesis in RA, which is related to the up-regulation of the expression of phosphate and tension homelessness deleted on chromoten (PTEN) to inhibit PI3K/Akt signal activation, the restoration of dynamic balance of pro/anti-angiogenic factors in vascular endothelial cells (VEC), and the reduction of FLSs stimulation to VEC by inhibiting the VEGF/SPK1/S1P pathway. Another study (107) showed that GE induced apoptosis in FLSs of AIA rats by regulating the expression of apoptosis-related genes and inhibiting the activation of ERK signals. The above studies have demonstrated that GE has anti-inflammatory, immunomodulatory, anti-angiogenic and pro-apoptotic effects on FLSs in RA, and it can be used as a potential therapeutic agent for RA.

### Andrographolide

Andrographolide (AD) is the main active diterpenoid alkane separated from the leaf extract of Andrographis paniculata (Burm. F.) Nees. It has many pharmacological effects, such as anti-inflammation, anti-oxidation, anti-angiogenesis and protecting liver. At the same time, it also has certain anti-arthritis effect (108). The preliminary study (109) showed that AD could inhibit cell growth and promote apoptosis of RA-FLSs. Li et al. found that (110), AD can reduce the production of anti-CII, TNFα, IL-1β and IL-6 in the serum of CIA mice, and can also decrease the phosphorylation of p38 MAPK and ERK1/2 in RASFs induced by TNFα in a dose-dependent manner. This suggests that AD may play an anti-inflammatory role in RA by inhibiting the activation of MAPK pathway. Further studies (111, 112) have shown that AD can treat arthritis and systemic inflammation in rats with RA by regulating oxidative stress (inhibition of MDA and nitrite/nitrate levels, enhancement of antioxidant enzymes SOD, CAT and GSH activities), reduction of chemokines and inflammatory factors (CXC chemokine ligand2, TNF-α, IL-6), reduction of neutrophil aggregation and infiltration and NetOS, and promotion of neutrophil apoptosis. Another study (113) has also shown that AD can attenuate hypoxia-induced migration and invasion of RA-FLS and the expression of MMPs (MMP-1, MMP-3 and MMP-9) by inhibiting the HIF-1α signaling pathway. These studies suggest that AD has anti-RA activity and can be used as a potential therapeutic agent for RA.

### Artesunate

Studies (114, 115) have found that the antimalarial drug artemisinin analog artesunate (ARS) has strong immunosuppressive activity in RA model, which can inhibit the formation of germinal centers, B cell proliferation and the production of autoantibodies, increase Foxp3 expression, reduce the formation of pannus, and cartilage and bone erosion. ARS is also an effective antioxidant (116), which is able to inhibit the production of ROS in osteoclast precursor cells and maintain oxidative homeostasis, thereby inhibiting osteoclastogenesis, by activating p62/Nrf2 signaling pathway and inhibiting NFATc1 signaling. The research by He et al. (117) has demonstrated that ARS may also have anti-angiogenic effect and play a role in RA-FLS. ARS may reduce the secretion of VEGF and IL-8 induced by TNFα or hypoxia, as well as the expression of HIF-1α, possibly by inhibiting the activation of PI3K/AKT. It has been reported (118–121) that ARS can inhibit the proliferation of chondrocytes and accelerate apoptosis and autophagy in RA rats, inhibit the migration and invasion of RA-FLSs and reduce the secretion of IL-1β, IL-6 and IL-8, alleviate inflammatory symptoms and prevent the destruction of cartilage and bone, which is related to the inhibition of NF-kB signaling pathway, the activation of PI3K/AKT/mTOR signaling pathway, and the phosphorylation of p90 ribosomal kinase 2 (RSK2). These studies have shown that ARS has a significant anti-arthritis effect in RA and can be used as adjuvant treatment for patients with RA.

## Glycoside

### Ginsenoside

Ginsenoside, an active ingredient of triterpenoid saponins in Panax ginsengC. A. Mey, has many effects such as anti-tumor, anti-oxidation, anti-inflammation, anti-fatigue and improving immunity (122). A study (123) has shown that ginsenoside Rg1, Rg3, Rg5, Rb1, Rh2 and CK have anti-RA effect, and ginsenoside CK has the strongest effect on RA, with strong anti-inflammation and immune regulation. Ginsenoside compound K (GCK) is the main degradation product of oral ginsenoside in the human intestinal tract and can play an anti-arthritis role from three aspects of anti-inflammation, immune regulation and bone protection, respectively (124). The research by Choi et al. (125) showed that GCK could inhibit osteoclast formation, which might be related to the molecular mechanism through inhibiting the JNK and ERK pathways, reducing the expressions of MMP-1, MMP-3 and RANKL in RA-FLS, and inhibiting RANKL-induced IκBα degradation and NFATc1, thereby inhibiting TRAP^+^ osteoclast-like cell formation. Chen et al. (126) showed that GCK could alleviate arthritis in AIA rats by inhibiting T cell activation, and the mechanism was through inhibiting T cell proliferation, CD25 expression and IL-2 production, as well as up-regulating immature T cells and Treg cells in the spleen, thus exerting immunomodulatory effects on RA model to improve arthritis. Their further research showed (127) that GCK could inhibit the abnormal activation and differentiation of T cells in CIA and AIA animal models, and its potential molecular mechanism might be the inhibition of CCL21/CCR7-mediated DC migration and signal transduction between T cells and DC. Their research also showed (128) that GCK could increase the level of serum antibodies (IgG1, IgG2a and anti-type II collagen) in CIA mouse model, promote the proliferation of B cells and restore B cell subsets. It could also promote the endocytosis of IgD-BCR by enhancing the expression of β-arrestin1 and promoting the co-location between IgD and β-arrestin1, thus inhibiting the activation of B cells. GCK also exerts joint protection by inhibiting the proliferation, migration and secretion of synovial cells (129), whose molecular mechanism is to inhibit the secretion of TNF-α in AIA-FLS rats, down-regulate the expression of tumor necrosis factor receptor type 2 (TNFR2), and inhibit TNF-α-mediated proliferation, migration and secretion of AIA -FLS. These studies show that GCK can treat RA in many ways, and it is an effective drug for treating RA. Compared with GCK and methotrexate (MTX) alone in treating AIA rats, GCK combined with MTX has better curative effect, and can also reduce anemia (130). In addition, the anti-RA mechanism of ginsenoside Rg1 may be related to its anti-inflammatory effect by up-regulating peroxisome proliferators activated receptor-γ (PPAR-γ) and then inhibiting the NF-κB signaling pathway (131). The anti-RA mechanism of ginsenoside Rg3 is related to its efficacy in regulating the oxidative phosphorylation pathway, and enhancing the ability of CD4^+^ CD25^+^ Foxp3^+^ Treg cells to maintain peripheral immune tolerance, thereby resisting inflammation and immunosuppression (132). In conclusion, ginsenoside (especially GCK), as a star natural product that can be used for the treatment of various diseases, may also be an effective drug for the treatment of RA.

### Paeoniflorin

Paeoniflorin, a monoterpenoid glycoside compound, is the main active ingredient of total glucosides of paeony widely used in the treatment of RA in China, and has multiple biological effects such as anti-inflammation, anti-oxidation, anti-thrombosis, anti-depression, anti-tumor and immune regulation (133). It has been reported (134–136) that TGP/paeoniflorin can exert anti-RA effects by inhibiting inflammatory processes, inhibiting lymphocyte activation, inhibiting the proliferation and differentiation of synovial cells, and preventing the formation of new blood vessels and the production of MMPs. Jia et al. showed (137) that paeoniflorin can improve arthritis of RA model rats by anti-oxidative stress, anti-inflammatory and reducing COX-2 expression. Paeoniflorin decreased the concentration of MDA in serum, increased the activities of antioxidant enzymes (SOD, CAT and GSH-Px), and also decreased the activities of inflammatory factors (NF-κB p65, TNF-α, IL-1β and IL-6) and the expression level of COX-2 protein. Zhu et al. (138) proved that paeoniflorin could significantly improve the symptoms of CIA rats, reduce the levels of pro-inflammatory factors (TNF-α, IL-1β and IL-6), and down-regulate the expression of p-NF-κB p65 and p-myosin-binding subunit (MYPT1). P-NF-κB p65 was a marker of NF-κB activation, and p-MYPT1 was a commonly used indicator of Rho-associated protein kinase (ROCK) activation. ROCK up-regulates the inflammatory gene by regulating NF-κB activity and mediates the development of RA. All these have suggested that paeoniflorin might improve RA by inhibiting inflammation mediated by the ROCK/NF-κB signaling pathway. The research by Xu et al. (139) showed that paeoniflorin could reduce bone destruction and inflammatory infiltration of joints in CIA mice and inhibit osteoclast differentiation. The mechanism of inhibiting osteoclast differentiation might be realized by inhibiting NF-κB signaling pathway to reduce the expression of osteoclast-specific genes (TRAP, cathepsin, and MMP-9). In addition, paeoniflorin can also inhibit the proliferation, migration, invasion and inflammation of RA-FLS and accelerate cell cycle arrest by regulating the circ-FAM120A/miR-671-5p/MDM4 axis (140). These studies have shown that paeoniflorin can be used as an effective drug for the treatment of RA.

### Paeoniflorin-6 ‘-o-benzenesulfonate

Paeoniflorin-6 ‘-o-benzenesulfonate (CP-25) is a novel ester derivative of paeoniflorin, which has better anti-inflammatory and immune regulation effects than paeoniflorin (141). Wei’s research group found (142) that CP-25 had anti-inflammatory and immunomodulatory effects on AIA rats, and was able to regulate the expression of B cell activator (BAFF)/BAFF-R in CD4^+^ T cells to inhibit the growth of AIA-FLS co-cultured with BAFF-activated CD4^+^ T cells and the secretion of cytokines. Subsequent studies (143) showed that CP-25 could reduce CIA in mice and regulate B cell function by down-regulating the BAFF/BAFF-R-mediated BAFF-TRAF2-NF-κB signaling pathway. CP-25 can improve the ankle pannus formation in AA rats (144), and its possible mechanism is that it inhibits the plasma membrane localization of G protein-coupled receptor kinase 2 (GRK2) in human umbilical vein endothelial cells (HUVECs) and down-regulates the GRK2-induced endothelial C-X-C chemokine receptor type 4 (CXCR4)-ERK1/2 signaling pathway to exert anti-angiogenic effects. Jia et al. research found (145) that, CP-25 inhibited the proliferation of FLS and the secretion of PGE2 and TNF-α in AIA rats, and promoted the up-regulation of EP4 receptor and the down-regulation of GRK2 in AIA-FLS. This suggests that CP-25 blocked the progression of AIA in rats by regulating the expression of GRK2/EP4 receptors, correcting immune function and inhibiting the proliferation of abnormal FLS. Further study found (146) that CP-25 can directly target GRK2 and down-regulate the interaction between GRK2 and EP4, which may be achieved by controlling the key amino acid residue Ala321 of GRK2. The above studies have shown that CP-25 has a good anti-RA effect, and GRK2 may be a target for its treatment of RA.

## Other

Natural medicines with anti-RA activity are concentrated in polyphenols, flavonoids, alkaloids, terpenes (including sesquiterpenes, monoterpenes, diterpenoids and triterpenes), and glycosides (including saponins, glycosides, cardiac glycosides and iridoid glycosides). In addition to the above, some natural medicines have been proved to have anti-RA activity (**Table 1**): polyphenols including curcumin, chlorogenic acid and punicalagen, flavonoids including hesperidin, 7,3’-dimethoxyhesperidin, kaempferol and genistein, terpenes including pentaacetyl geniposide, gentiopicrin and betulinic acid, glycosides including emodin, and cyanidin-3-O-glucuronide, alkaloids including berberine and matrine, α-mangiferin (a xanthone derivative of mangosteen pericarp), cinnamaldehyde (an aldehyde active compound Chinese medicine herb Cinnamomum cassia), thymoquinone (the main component of the volatile oil in Nigella sativa L.), periplocin (one of the main active components in Periploca sepium Bunge).

**Table 1:** Other Natural medicines of targeted RA and their mechanism of action.

Natural medicines	Model/Cell	Dosage	Mechanism of action	Ref.
Curcumin	CIA rats FLSs	200 mg/kg 5, 10, 20 ng/ml	MAPK, ERK1/2, AP-1, mTOR and NF-kB↓	(147–149)
Chlorogenic acid	FLSs CFA rats	50, 100 mmol/L 5, 25, 50, 100 mg/kg	activation of JAK/STAT and NF-κB pathway↓. IL‐17/IL‐17RA/STAT‐3 cascade pathway↓, TLR‐3, IL‐23, GM‐CSF, Cyr61, RANKL↓	(150–152)
Hesperidin	AIA mice	20 mg/kg	PI3K/AKT signaling pathway↓, levels of MMP3, MMP9, and MMP13 in FLSs↓, the polarization of macrophages to M1↓	(153)
7,3 ′-dimethoxyhesperidin	AIA rats	20,40, 80 mg/kg	activation of JAK2/STAT3 pathway↓, regulate the expression of Bcl-2/Bax	(154, 155)
Kaempferol	CIA mice FLSs	100, 200 mg/kg 2, 5, 10, 20, 40 μM	activation of NF-κB and MAPK pathway↓, AKT/mTOR pathways↓, bFGF-induced FGFR3-RSK2 signaling pathway↓	(156, 157)
Matrine	CIA rats CIA FLSs	100 mg/kg 0.75 mg/ml	NF-κB pathway↓, regulate the imbalance of Th1/Th2 cytokine response, activation of JAK/STAT pathway↓	(158, 159)
Berberine	CIA rats	75, 150 mg/kg	regulate the PI3K/Akt, Wnt1/β-catenin, AMPK/lipogenesis and LPA/LPA_1_/ERK/p38 MAPK pathways, regulate the balance between Treg/Th17 cells, DC activation↓	(160, 161)
Pentaacetyl geniposide	AIA FLSs MH7A	50, 100, 200 μM 12.5, 25, 50 μM	activation of NF-κB and Wnt/β-catenin pathway↓	(162, 163)
Gentiopicrin	HFLS AIA rats RA-FLS	5-25 μM 100, 200 mg/kg 50, 100 μM	p38 MAPK/NF-κB pathway and the ROS-NF-κB-NLRP3 axis↓	(164, 165)
Betulinic acid	AIA rats RA-FLS CIA mice	20, 40 mg/kg 2.5, 5, 10, 20 μM 20 mg/kg	Rho/ROCK signaling pathway↓, block the activation of AKT/NF-κB pathway and NF-κB nuclear accumulation↓	(166–168)
Emodin	CIA mice AIA mice	10 mg/kg 30 μg/kg	NF-κB pathway↓, neutrophil apoptosis↑, neutrophil autophagy and NETosis↓	(169, 170)
α-mangiferin	AIA rats RA‐FLS	40 mg/kg 10, 50, 100 μM	the polarization of M1 macrophages↓, activate CAP, SIRT1↑, PPAR-γ↑, ROS production and ERK1/2 phosphorylation↑	(171, 172)
Cinnamaldehyde	MH7A CFA rats CFA FLSs	40, 60, 80 nM 20 mg/kg, 20 μM	JAK/STAT and PI3K/AKT pathway↓	(173, 174)
Thymoquinone	RAW 264.7 RA-FLS	2.5, 5, 7.5, 10 μM 1, 5 μM	RANKL-induced activation of NF-KB and MAPKs signals and ROS production↓, ASK1-p38/JNK pathway↓	(175, 176)
Cyanidin-3-O-Glucoside	CIA mice FLS, RASF, MNCs	25, 50 mg/kg 10, 20, 40 μM	activation of NF-κB and MAPK signaling pathways↓, relieve inhibition of CD38^+^ NK cells on Treg cell differentiation	(177, 178)
Genistein	MH7A RA-FLS	15, 20, 25 μmol/L 37 μM	JAK2/STAT3/VEGF pathway↓, Erk1/2-mediated RA-FLS proliferation and EGF-induced MMP-9↓	(179, 180)
Punicalagin	RA-FLS, CIA mice	12.5, 25, 50 μM 10, 20, 50 mg/kg	block the activation of NF-κB↓, M1 phenotypic polarization and focal ptosis↓	(181, 182)
Periplocin	AIA rats RA-FLSs	50 mg/kg 10, 20, 30 μM	*T-bet*, *GATA3*, and *C-Jun* genes↓, cleaved caspase-3 and caspase-9↑, regulate the expression of Bcl-2/Bax, NF-κB pathway↓	(183, 184)

↑, increase, up-regulate, promote or improve; ↓, suppress, down-regulate, reduce, or inhibit; bFGF, basic fibroblast growth factor; RSK2, p90 ribosomal S6 kinase 2; LPA, lysophosphatidic acid; CAP, Cholinergic anti-inflammatory pathway; ASK1, apoptosis-regulated signaling kinase 1; RA-FLS, fibroblast-like synoviocytes from human RA patients; CIA FLSs, FLSs from CIA rats; MNCs, mononuclear cells; RASFs, RA synovial fibroblasts; GM‐CSF, granulocyte-macrophage colony stimulating factor; Cyr61, Cysteine-rich angiogenesis inducer 61; T-bet, T-box transcription factor; GATA3, GATA binding protein 3.

## Mechanism of action against RA

### Anti-inflammation and anti- oxidation

COX-2, a key mediator in the inflammatory process, catalyzes the hydrolysis of glycerophospholipids to release arachidonic acid, which is converted to PGs, including prostacyclin, thromboxane A2, PGE2 and PGD2 during inflammation (185). Among them, PGE2 is a lipid signaling molecule involved in pain and inflammation, which is involved in various pathological processes and overproduced in patients with RA. Clinically, non-steroidal anti-inflammatory drugs (NSAIDs) for the treatment of RA are achieved by inhibiting COX to inhibit the expression of PGE2. The selective COX-2 inhibitors are a new class of NSAIDs, such as etodolac (186) and indomethacin (187). The synovial tissues and fluids of patients with RA have high levels of MMP-1, MMP-3, MMP-9 and MMP-13, which play a key role in the degradation of connective tissue components in RA cartilage (156, 188). The migration and invasion of RA-FLS cells may be mediated by the production of MMP-2 and MMP-9, and exogenous MMP-9 pretreatment can reverse the inhibitory effect of artesunate on the invasion of RA-FLS (119). MMPs are the main proteases involved in the invasion and degradation of the anatomical barrier. They play a key role in cartilage destruction of inflamed joints by assisting RASFs in attacking the microvascular basement membrane and stroma and destroying the extracellular matrix of the joint structure in RA (189). Therefore, reducing the production of MMPs, inhibiting the activation of MMPs or increasing the production of endogenous inhibitors of MMPs are feasible strategies for the treatment of RA. It is known that cytokines play a key role in the pathogenesis of RA. For example, TNF-α, IL-1β, IL-6, IL-8 and IL-17A can stimulate inflammatory reactions in arthritic joints and synovial tissues (3). Among them, IL-1β and TNF-α can enhance the expression of COX-2, PGE2 and MMPs in human RASFs (158, 188, 190), and play an important role in the development of RA. In recent years, targeted therapy has become a hot research topic and a new therapeutic approach in the treatment of RA, and the cytokines TNF-α and IL-6 have become mature targets in the treatment of RA (191). IL-6 is also a major pro-inflammatory factor in the pathogenesis of RA, but FLSs does not express membrane IL-6 receptors, and transmits IL-6 pro-inflammatory signals mainly through the connection of IL-6 with sIL-6R, which is present in RA joints (192).

**Figure 1:**
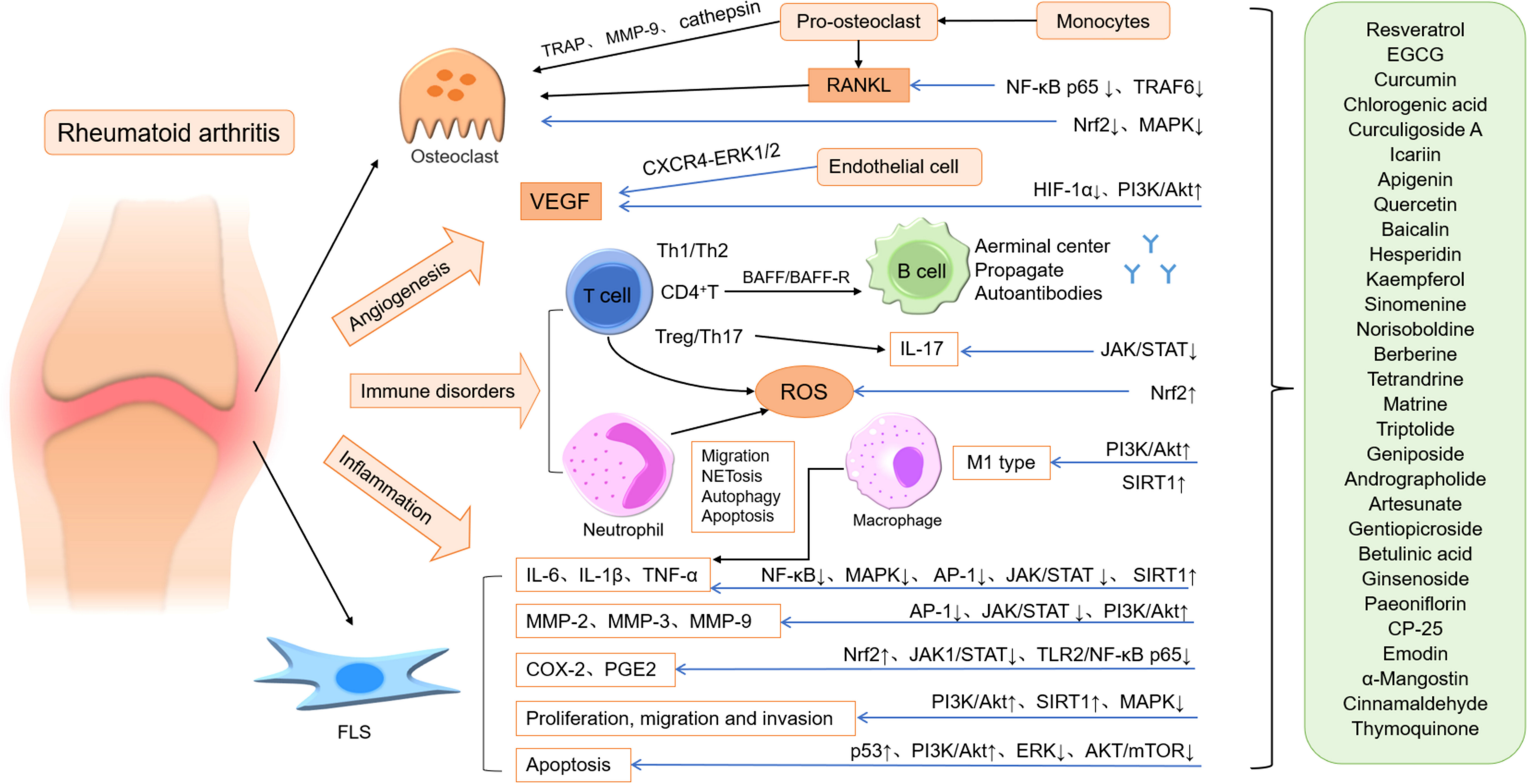
The main mechanism of natural medicines acting on RA. ↑: increase, promote or up regulation; ↓: decrease, down regulation or inhibit; Blue arrow: inhibit.

ROS production, lipid peroxidation, protein oxidation and DNA damage in RA patients are increased, and the activity of antioxidant defense system is decreased, resulting in oxidative stress (193). Chronic oxidative stress in synovial T-lymphocytes of patients with RA results from intracellular ROS production (mainly H_2_O_2_) (194), and ROS may be involved in destructive pathological events of RA. A recent study has shown that fatty acid oxidation (FAO) metabolism is involved in bone destruction in RA patients (195). Selegiline was able to improve the progression of RA (196), possibly by reducing the decomposition of catecholamines in synovial fluid to reduce H_2_O_2_ production and inhibit pro-inflammatory cytokines in situ. Oxidative stress can promote the production of inflammatory cytokines, and free radicals can react with protein, lipids, nucleic acids and other cellular macromolecules, playing an important role in the pathogenesis of RA (197). Considering the important roles of inflammation and oxidative stress in the pathogenesis of RA, the researchers focused on the treatment of RA with new drugs with potent anti-inflammatory and antioxidant activities. Many natural medicines, such as resveratrol, EGCG, hesperidin, CA, sinomenine, AD, ARS, and gentiopicroside, have significant anti-inflammatory and antioxidant effects on RA (**Figure 1**).

### Immunoregulation

RA is a common autoimmune disease. Patients with RA have immune system dysfunction, and immune dysfunction is one of the important factors for the development of RA. Adaptive immunity plays a central role in the pathogenesis of RA. Synovial T cells are involved in the induction of antibody production and local inflammation (198). In particular, CD4^+^ T cell subsets can differentiate to produce a variety of pro-inflammatory cytokines and chemokines, which are involved in the pathogenesis of RA. The epigenetic stability of Treg is unstable. CD4^+^ CD25^+^ Foxp3^+^ Tregs cell dysfunction is common in autoimmune diseases (199), and it may be converted into pathogenic Th17 cells after repeated amplification (200). Foxp3 is the main regulator of immunosuppression of Treg cells. One of the strategies for the treatment of RA is to convert traditional Treg cells into Foxp3^+^ Treg cells with stable inhibitory function, and reduce the related inflammation caused by the conversion of Treg cells into Th17 cells (199, 200). B-cell infiltration in the synovial membrane, especially new B-cell infiltration of ectopic lymph, is associated with the severity of RA. Part of B cells in the synovial membrane differentiate into plasma cells to produce autoantibodies such as anti-citrulline protein antibody (ACPA), and the other part differentiate into effector B cells to produce proinflammatory cytokines, including IL-1, IL-6, IL-12, TNF-α, and express RANKL. RANKL can promote osteoclastogenesis and participate in bone and joint destruction of RA (201). BAFF is necessary for the growth and development of B cells. B-cell function was regulated by inhibiting the activation of BAFF receptors, thereby reducing CIA inflammation in mice.

Innate immunity also plays an important role in the pathological development of RA. Synovial macrophages, which are directly involved in the formation of RA synovitis and joint destruction, are the core target cells of RA (202). Activated macrophages (M1 type macrophages) invade the synovium, and the imbalanced ratio of M1 type and anti-inflammatory macrophages (M2 type) in synovial tissues as well as the differentiation of macrophage precursor cells into osteoclasts are important characteristics of RA (203). Zheng et al. (204) prepared IL-10 pDNA/DSP-NPs that could actively target macrophages in synovial tissues, which had good therapeutic effects on CIA rats. IL-10 inhibits the inflammatory response by down-regulating the synthesis and expression of pro-inflammatory cytokines in macrophages and promoting M1-M2 polarization of macrophages. This indicates that inhibiting the M1 polarization of macrophages is a feasible measure for the treatment of RA. NET and NETosis are involved in the pathological process of autoimmune diseases such as RA. The extracellular citrulline autoantigen present in the joints of RA patients is mainly derived from the increased NET, which can cause the inflammatory response of synovial fibroblasts in patients and release a series of inflammatory mediators (such as pro-inflammatory cytokines, adhesion molecules and chemokines) to drive the progression of RA (205). Some natural medicines, such as andrographolide, emodin and triptolide, can improve RA by inhibiting inflammatory function of neutrophils, and inhibiting NETosis, autophagy and migration. Nowadays, neutrophils are also considered as potential targets for the treatment of RA.

The existence of autoantibodies is a key factor in RA. The detection of RF and ACPA is extremely important in the diagnosis and classification of RA. ACPA can stimulate the release of macrophages and FLSs cytokines, leading to the development of inflammation (201, 206). Patients with RA have been found to have significantly elevated serum IgG4 levels compared to the general population (207). RF recognizes the Fc domain of IgG4 and forms the RF-IgG4 immune complex, which may activate the complement system and cause synovial damage, with a pathogenic effect in RA. IgG4 is likely to be an active biomarker of RA.

### Main related signaling pathways and transcription factors of RA

The activation of synovial cell TLR leads to exacerbation of arthritis (208), in which the expression of TLR2, TLR3, TLR4 and TLR5 in RA synovial cells is increased and they are involved in the regulation of inflammatory factor production in RA synovial cells. TLR-dependent MAPK signaling pathway is a key pathway for mediating the occurrence of RA (209). Studies have shown that NF-κB and MAPKs (three major kinases: JNK, p38 and ERK) are expressed in cultured RA-FLSs and easily activated by IL-1β and TNF-α (58, 59), involving in RA inflammation and joint destruction. Inhibition of activation or downregulation of MAPK signaling pathway weakens the inflammatory response and oxidative stress in RA model, inhibits the proliferation, migration and invasion of FLSs, regulates the body immunity, and inhibits osteoclastogenesis. The transcription factor NF-κB is an important regulator of immune and inflammatory responses, and NF-κB p65-mediated increased trans-activation plays a key role in the pathogenesis of chronic inflammatory diseases (210). The beneficial effects (down-regulation of inflammatory factors) of some natural compounds (such as sinomenine, tetrandrine and paeoniflorin) in RA are attributed to the reduction of NF-κB p65 signal. The above studies have shown that down-regulation of RANKL inhibits osteoclastogenesis and reduces bone loss and joint destruction, which is related to the inhibition of NF-κB signal activation. The reduction of bone erosion and osteoclast differentiation by NOR is associated with inhibition of nuclear translocation of NF-κB p65. Therefore, targeted p65 activation may also be a feasible strategy for RA treatment in the future.

HIF is a transcription factor that responds to cellular oxygen supply. As one of the important mediators of RA, it can induce angiogenesis, promote FLSs migration and cartilage destruction, and inhibit the apoptosis of synovial cells (211). HIF-1α can regulate the expression of VEGF gene under hypoxic stimulation (212), which is related to the adaptation of RA synovial membrane to the hypoxic microenvironment (211). The down-regulation of HIF-1α significantly reduces synovitis and angiogenesis, which may be a potential therapeutic target for RA (213). Transcription activator AP-1 (a heterodimer consisting of c-Fos and c-Jun) and Nrf2 are also redox-sensitive transcription factors, which are closely related to the pathogenesis of RA (214). In RA, AP-1 regulates cytokine and MMP production (215), which are important in arthritis. C-Fos/AP-1 can trans-activate *MMP* gene, thus mediating the degradation of cartilage and bone matrix (216). In addition, c-Fos/AP-1 and IL-1β affect each other’s gene expression and activity to drive osteoclasts and interact with osteoclasts to promote joint destruction (216). Nrf2, involved in osteoclastogenesis, PGs secretion and ROS production (62, 116), is very important for regulating oxidative stress, inflammation, immune response and cartilage and bone metabolism (217), and serves as an important target for inflammatory disturbance and oxidative stress in RA. Nrf2 binds to antioxidant response elements (ARE) and oxidative stress-related proteins (including GSH, HO-1, and oxidoreductase I), and then scavenges cytotoxic electrophiles together with ROS (218). Activation of Nrf2-ARE may inhibit the production or expression of pro-inflammatory mediators to reduce early inflammation-mediated tissue damage (219). In addition, activation of Nrf2 leads to the synthesis of HO-1 and the formation of a large number of bioactive metabolites, which may control the activation and metabolism of joint cells and play a regulatory role in joint destruction (220). Targeted Nrf2 may be an effective treatment.

The JAK/STAT signaling pathway, as the main target for inhibiting the action of a variety of cytokines (221), is involved in the signal transduction of many key cytokines for the pathogenesis of RA and has been considered as a potential target for the treatment of RA (222). After the effector molecules of JAK (such as cytokines, IFN, colony-stimulating factors, and growth factors) bind to type I and type II receptors (these receptors consist of various subunits, each of which is associated with a JAK molecule), JAK is activated and provides a docking site for STAT molecule by transferring phosphate to the tyrosine residues of receptor subunits through autophosphorylation, followed by STATs phosphorylation-mediated signal transduction of the nucleus (223). Inhibiting the JAK/STAT pathway to inhibit Th17 differentiation and expression of inflammatory factors (TNF-α, IL-1β, IL-6, IL-17, etc.) in the RA model, promote FLSs apoptosis, inhibit FLSs inflammatory apoptosis, and inhibit the expression of MMP-2 and MMP-9. Triptolide inhibits IL-6/sIL-6R complex-induced FLSs proliferation and inflammatory cytokine expression by inhibiting the JAK2/STAT3 signaling pathway. Studies have shown that both classical and trans IL-6 signals can trigger STAT3 phosphorylation and activate the JAK/STAT3 pathway in a time-dependent manner (224). However, the IL-6 trans signal results in more intense STAT3 phosphorylation than classical signals. JAK inhibitor is a disease-modified anti-rheumatic drug targeting JAK. It can interfere with JAK signal transduction and STAT signaling pathway by inhibiting the activity of one or more JAKs, and simultaneously inhibit the production of multiple cytokines to reduce inflammation and inflammation-related pain (225, 226).

Elevated levels of pro-inflammatory cytokines are considered to be an important factor in the development of RA. Some pro-inflammatory cytokines (such as TNF-α, IL-1β, IL-6, and IL-34) can activate a variety of signal transduction pathways, including NF-κB, MAPK, JAK/STAT and PI3K/Akt pathways. The activated signaling pathways promote the release of pro-inflammatory cytokines, leading to further aggravation of RA (227, 228). PI3K/Akt signaling pathway is involved in the regulation of many basic cellular processes, including cell growth, transcription, translation, proliferation, motility, and glycogen metabolism. Under the stimulation of growth factors, PI3Ks are recruited onto the plasma membrane to catalyze the phosphorylation of the 3-hydroxy group of PIP2 to generate PIP3, which then acts as the second messenger, recruiting PDK1 and AKT proteins onto the plasma membrane to partially activate AKT by PDK1. The activated AKT will further activate the downstream regulatory pathway (the downstream target is mTOR), and the conversion process can be reversed by PTEN (a negative regulatory factor of PI3K) through dephosphorylation of PIP3 (229). PI3Ks are involved in a wide range of intracellular regulatory mechanisms, and their functional deregulation has been associated with a variety of human diseases (230). Studies have shown that the PI3K/Akt signaling pathway plays an important role in multiple mechanisms of RA development, including angiogenesis, the proliferation, apoptosis and metastasis of FLS, and the expression of MMPs (43, 117, 153, 173), and it can also be considered as a potential target for the treatment of RA.

SIRT1, a nicotinamide adenine dinucleotide (NAD)-dependent histone deacetylase, is able to regulate inflammation, oxidative stress, mitochondrial function, immune response, cell differentiation, proliferation, and metabolism, and its dysfunction may participate in the development of autoimmune diseases (231). In recent years, there are more and more studies on the regulation of energy metabolism and immune function of SIRT1 in RA (232). Moreover, SIRT1 can positively affect cartilage by promoting the survival of chondrocytes under stress conditions (233). Studies (14, 234) have shown increased expression levels of SIRT1 in serum from RA patients or in FLS and chondrocytes from CIA mice. A recent study (235) has shown that some variants of the SIRT1 gene (rs3740051, rs7069102, and rs1467568) are associated with RA susceptibility in the Chinese Han population. In RA model, some natural medicines (such as resveratrol, baicalin and α-mangiferin) antagonize the transcriptional activity of NF-κB by up-regulating the expression of SIRT1, thereby inhibiting the expression of a variety of inflammatory cytokines (such as TNF, IL-6 and IL-1β), and inhibiting the proliferation, invasion and migration of FLSs, which has a positive effect on RA. Therefore, SIRT1 may be a new target for the treatment of RA.

## Discussion

For thousands of years, Chinese herbal medicine has played a pivotal role in maintaining human health and treating various diseases. At present, Chinese herbal medicine is considered to be an important source for screening and seeking new candidate drugs, and natural medicines derived from Chinese herbal medicine have received more and more attention at home and abroad. Similarly, Chinese herbal medicines that have played an active and significant role in the treatment of rheumatic diseases such as RA, such as Radix Gentianae Macrophyllae, Radix Stephaniae Tetrandrae, Caulis Sinomenii, and Caulis Lonicerae, have also become the targets for finding and screening new potential therapeutic agents for RA. Studies have also proved that the active ingredients in these Chinese herbal medicines (gentiopicroside, tetrandrine, sinomenine and chlorogenic acid) do have anti-RA potential.

In this paper, the natural medicines with obviogentiamarinus anti-RA activity were reviewed. These natural medicines are mainly classified as: flavonoids, polyphenols, alkaloids, glycosides and terpenes. Reducing the expression of inflammatory mediators, immune regulation, anti-oxidative stress, preventing angiogenesis, inhibiting the proliferation and migration of FLSs, and inhibiting the differentiation of osteoclasts are all important mechanisms of natural medicines in the treatment of RA. The main related signaling pathways and transformation factors are summarized as follows: NF-κB, MAPK signaling pathway, JAK2/STAT3 signaling pathway, PI3K/Akt signaling pathway, AP-1, Nrf2, SIRT1, and HIF-1α. Some natural medicines, such as curcumin, sinomenine, andrographolide, emodin and GCK, can get better therapeutic effects and/or less side effects when being used in combination with MTX for the treatment of RA. These fully show the advantages of natural drugs in the treatment of RA: multi-channel, multi-target, natural and effective, with little toxic and side effects.

At present, there are four kinds of western medicines used clinically to treat RA: nonsteroidal anti-inflammatory drugs, glucocorticoids, anti-rheumatic drugs and biological agents. However, these drugs are not satisfied with the treatment effect because of their big toxic and side effects, poor tolerance and compliance of patients, high price and other reasons. MTX is the most commonly used anti-rheumatic drug, and it is often used in combination with other drugs (glucocorticoids or non-steroidal anti-inflammatory drugs) to achieve better therapeutic effects. From the research and development in recent years, it is an important development trend to make full use of the advantages of natural medicine and combine western medicine to treat RA with considerable effect.

At present, there are few kinds of natural medicines for clinical treatment of RA, and most of them are in the stage of preclinical research. Sinomenine, tripterygium glycosides and total glucosides of paeony have been clinically used for the treatment of RA. Resveratrol, a star natural medicine, has been found to have beneficial effects on the treatment of a variety of diseases, showing great therapeutic potential in the study of RA. Triptolide, the main active ingredient of tripterygium glycosides tablet, has significant curative effect in the treatment of RA due to its anti-inflammatory and immunosuppressive effects. However, its toxic and side effects, mainly gastrointestinal adverse reactions and reproductive toxicity, will also affect the liver and kidney function and cardiovascular system to a certain extent, which is still a thorny problem. In order to improve the therapeutic effect of natural medicines, the research and development of natural medicines derivatives are also one of the current hot spots. Compared with the parent natural medicines, their derivatives have attracted much attention due to their stronger pharmacological effects. For example, hesperidin and its derivative 7,3’-dimethoxyhesperidin, geniposide and its derivative pentaacetyl geniposide, paeoniflorin and its derivative paeoniflorin-6 ‘-o-benzenesulfonate.

Therefore, finding effective and low-toxic active substances from traditional Chinese medicines for treating rheumatoid diseases, or developing more efficient and quality-controlled drugs based on them, is also an important development direction for treating RA at present and in the future. In the future, with more extensive and in-depth research and clinical trials, it is hoped that natural medicines, including diet and herbs, will be more widely accepted, used alone or as adjuvant drugs for the treatment of RA.

## Author contributions

All authors listed have made a substantial, direct and intellectual contribution to the work, and approved it for publication.

## Funding

This work was supported by a grant from the National Natural Science Foundation of China (No. 81803991 and 81904166); Natural Science Foundation of Jiangsu Provincial (No. BK20130543); Open Project of Zhenjiang Medical Research Center for Traditional Chinese Medicine and Gynecology (No. SS202204-KFA01); Special grant program of National Postdoctoral Science Foundation of China (No. 2014T70490); First-class Fund of National Postdoctoral Science Foundation of China (No. 2013M540426); 2022 Jiangsu University College Students Innovation Training Program Project (No. 3201504100, 3201504108).

## Conflict of interest

The authors declare that the research was conducted in the absence of any commercial or financial relationships that could be construed as a potential conflict of interest.

## Publisher’s note

All claims expressed in this article are solely those of the authors and do not necessarily represent those of their affiliated organizations, or those of the publisher, the editors and the reviewers. Any product that may be evaluated in this article, or claim that may be made by its manufacturer, is not guaranteed or endorsed by the publisher.
